# Functional Characterization of Sugar Beet M14 Antioxidant Enzymes in Plant Salt Stress Tolerance

**DOI:** 10.3390/antiox12010057

**Published:** 2022-12-27

**Authors:** Jinna Li, Bing Yu, Chunquan Ma, Hongli Li, Desheng Jiang, Jingdong Nan, Meng Xu, He Liu, Sixue Chen, Huizi Duanmu, Haiying Li

**Affiliations:** 1 School of Chemistry and Materials Science, Heilongjiang University, Harbin 150080, China; 2 Key Laboratory of Molecular Biology of Heilongjiang Province, College of Life Sciences, Heilongjiang University, Harbin 150080, China; 3 Engineering Research Center of Agricultural Microbiology Technology, Ministry of Education, Heilongjiang University, Harbin 150080, China; 4 Department of Biology, University of Mississippi, Oxford, MS 38677-1848, USA

**Keywords:** sugar beet M14 line, salt stress, antioxidant enzyme system, reactive oxygen species (ROS), ectopic expression

## Abstract

Salt stress can cause cellular dehydration, which induces oxidative stress by increasing the production of reactive oxygen species (ROS) in plants. They may play signaling roles and cause structural damages to the cells. To overcome the negative impacts, the plant ROS scavenging system plays a vital role in maintaining the cellular redox homeostasis. The special sugar beet apomictic monosomic additional M14 line (*BvM14*) showed strong salt stress tolerance. Comparative proteomics revealed that six antioxidant enzymes (glycolate oxidase (GOX), peroxiredoxin (PrxR), thioredoxin (Trx), ascorbate peroxidase (APX), monodehydroascorbate reductase (MDHAR), and dehydroascorbate reductase3 (DHAR3)) in *BvM14* were responsive to salt stress. In this work, the full-length cDNAs of genes encoding these enzymes in the redox system were cloned from the *BvM14*. Ectopic expression of the six genes reduced the oxidative damage of transgenic plants by regulating the contents of hydrogen peroxide (H_2_O_2_), malondialdehyde (MDA), ascorbic acid (AsA), and glutathione (GSH), and thus enhanced the tolerance of transgenic plants to salt stress. This work has charecterized the roles that the antioxidant enzymes play in the *BvM14* response to salt stress and provided useful genetic resources for engineering and marker-based breeding of crops that are sensitive to salt stress.

## 1. Introduction

Wild sugar beet (*Beta corolliflora* Zoss.) has excellent characteristics of drought resistance, frost resistance, salt tolerance, cold tolerance, and apomixis. In the early stage of the study, diploid cultivated beet (*B. vulgaris* L.) and tetraploid wild sugar beet (*B. corolliflora* Zoss.) were crossed by distant hybridization. After obtaining allotriploid, they were further backcrossed with cultivated sugar beet, and the M14 with the wild sugar beet chromosome 9 (*BvM14*) was selected for apomixix and high salt tolerance. It is a rare germplasm for studying plant salt stress tolerance mechanisms [1,2,3,4,5].

Reactive oxygen species (ROS), including superoxide anions (O_2_^•−^), hydroxyl radicals (HO_2_^•^), hydrogen peroxide (H_2_O_2_), and singlet oxygen (^1^O_2_), play an important role in plant metabolism, signal transduction, photosynthesis regulation, bacterial defense, and cell apoptosis [6,7,8]. Salinity affects plants growth and development through osmotic stress, ion toxicity, overproduction of ROS, and oxidative stress [9]. It is known that chloroplasts, mitochondria, peroxisomes, apoplast, and plasma membranes are the main sites of cellular ROS generation [10]. Overproduction of ROS can lead to severe damage of protein, membrane lipid, DNA, and other cellular components [11]. To cope with this challenge, plants have antioxidative mechanisms that consist of enzymatic and non-enzymatic components to regulate ROS synthesis and scavenging. The antioxidant enzymes of ROS scavenging in plants mainly include superoxide dismutase (SOD), ascorbate perxidase (APX), catalase (CAT), glutathione peroxidase (GPX), and thioredoxin (Trx) [12,13,14]. Some antioxidative genes have been cloned from rice [12], *Arabidopsis* [15], maize [14], and soybean [16], while only partial sequences of peroxisome *APX* gene coding region and *GOX* gene have been obtained from salt-tolerant sugar beet [17]. However, there are few reports about the different roles of these enzymes under salt stress.

In the past years, the salt tolerance characteristics of *BvM14* have been well-studied [1,2,4,18,19,20]. The differentially expressed proteins (DEPs) under salt stress (0, 200, 400 mM) have been studied by iTRAQ LC-MS/MS. A total of 76 DEPs have been identified in leaves of the *BvM14*. These proteins involve photosynthesis, metabolism, protein synthesis, protein folding and degradation, stress and defense, cell structure, transcription, and transport processes. Among them, six main proteins (GOX, PrxR, Trx, APX, DHAR3, and MDHAR) of the antioxidant enzymes system changed most significantly in the ROS scavenging system under salt stress [4]. However, to the best of our knowledge, salt tolerance of the *GOX*, *PrxR*, *Trx*, *APX*, *DHAR3*, and *MDHAR* genes and their relationships have not been characterized in sugar beet M14.

In this work, we aimed to investigate the following related questions. What are the reactions that are directly responsible for these six genes in the antioxidant enzyme system during salt stress? What is the relationship between these six genes in antioxidant enzyme system under salt stress conditions? How important are the six genes in antioxidant enzyme system during salt stress? To answer these questions, molecular biological methods were used to evaluate the functions of the six major antioxidant enzymes in *BvM14* under salt stress [3,4]. We generated a complete set of single mutants for the six key genes in *Arabidopsis* and analyzed the function of the six genes under salt stress. Understanding the mechanism of ROS scavenging allows for a powerful strategy to enhance crop salt tolerance.

## 2. Materials and Methods

### 2.1. Plant Materials, and Growth Conditions

Sugar beet M14 seeds were initially sowed in vermiculite for seven days. Then, the seedlings were planted in Hoagland solution and kept at 23 °C, 450 μmol.m^−2^. s^−1^, and 14 h/10 h light/dark cycles [3,4]. *Arabidopsis thaliana* (Columubia 0) seeds were treated with 5% (*w*/*v*) NaClO for 6 min, rinsed with sterilized water to remove NaClO. *Arabidopsis* plants were grown in soil under controlled conditions (22 °C, 300 μmol m^−2^ s^−1^ and 16 h/8 h light/dark cycles) [21].

### 2.2. Isolation and Sequence Analysis of Genes

Total RNA was isolated and subjected to reverse transcription by using the SuperScriptTM III Reverse Transcriptase kit (Invitrogen, Carlsbad, CA, USA). The full-length coding regions were PCR-amplified from the sugar beet M14 cDNA with gene specific primers (Supplementary Table S1).

### 2.3. Quantitative Real-Time PCR Analysis

Quantitative real-time PCR analysis (qRT-PCR) analyses were performed using SYBR Premix ExTaqTM II Mix (TaKaRa, Shiga, Japan). *GAPDH* (glyceraldehyde-3-phosphate dehydrogenase, accession no. DQ355800) was used as a reference. The expression levels of all candidate genes were analyzed by the 2^−ΔΔCT^ CT method. The primers used for qRT-PCR were listed in Supplementary Table S1 [1,2,22].

### 2.4. Generation and Phenotypic Analyses of Transgenic A. thaliana

At present, the genetic transformation system in sugar beet is not successful, so the gene function research will be carried out in *A. thaliana*. Currently, our team is experimenting with various approaches to the genetic transformation system in sugar beet.

We obtained the mutants *gox*, *prxr*, *trx*, *apx*, *mdhar*, and *dhar3* from the Arabidopsis Biological Resource Center (ABRC), genotyping PCR was performed to identify the T-DNA insertion. To construct 35S::*BvM14-GOX*, 35S:: *BvM14-PrxR*, 35S:: *BvM14-Trx*, 35S::*APX*, 35S:: *BvM14-MDHAR*, and 35S:: *BvM14-DHAR3* in *Arabidopsis*, the open-reading frames (ORF) were cloned into pCAMBIA1305.1, and transformed into *Arabidopsis* as described [23]. The T0 *Arabidopsis* seeds were screened on MS plates [21] containing 30 mg/L hygromycin, and the survival seedlings were further verified by reverse transcription polymerase chain reaction (RT-PCR) analyses. Homozygous T3 generation plants were used for further analyses [21].

### 2.5. Quantification of Biomass, MDA Content, H_2_O_2_ Content, Na^+^ and K^+^ Content, Morphological Index, and Physiological Indicators

The root length was measured in Murashige and Skoog (MS) medium. The 7-day-old seedlings were transferred to MS medium containing 150 mM NaCl for salt treatment 10 days. Three biological replicates were carried out.

The fresh weight and dry weight and physiological indicators of leaves were measured in the soil. The one-month wild type (WT) and transgenic plants were treated with 150 mM NaCl for 7 days.

The MDA content was measured as described by Wang [21]. The contents of H_2_O_2_ were measured by H_2_O_2_ assay kit (Comin Botechnology, Suzhou, China), the contents of glutathione (GSH), and ascorbic acid (AsA) were determined using the GSH and AsA assay kits, respectively (Comin Botechnology, Suzhou, China). The activities of catalase (CAT), ascorbate peroxidase (APX), glycolate oxidase (GOX), peroxiredoxins (PrxR), and thioredoxins (Trx) were determined in WT and transgenic leaves using an assay kit (Comin Botechnology, Suzhou, China). The activities of dehydroascorbate reductase (DHAR) and monodehydroascorbate reductase (MDHAR) were determined using an ELISA assay kit (Mlbio, Shanghai, China).

### 2.6. Cis-Regulatory Elements(CREs) Analysis

The promoters of the six stress-responsive genes in *BvM14* were analyzed for putative *cis*-elements using available genomic sequences and our transcriptomic data [19]. In addition, the 2000 bp genomic sequences located on the 5′ upstream of the Transcriptional Start Site (TSS) of the six stress-responsive genes sequences were extracted and analyzed with PlantCARE (https://bioinformatics.psb.ugent.be/webtools/plantcare/html/, accessed on 15 November 2022) and TB tools. The Promoter binding of transcription factors was predicted by PlantTFDB (http://planttfdb.gao-lab.org/prediction.php, accessed on 3 June 2022) analysis [16].

### 2.7. Statistical Analysis

For all the experiments, three biological replicates with three technical replicates of each treatment were measured. All data were analyzed using GraphPad Prism 8 (Dr. Harvey Motulsky, San Diego, CA, USA). For multiple comparions, one-way analysis of variance (ANOVA) was used to determine statistical significance among treatments at *p* < 0.05.

## 3. Results

### 3.1. Ectopic Expression of BvM14 Antioxidant Enzymes Enhanced Plant Salt Tolerance

**Figure 1:**
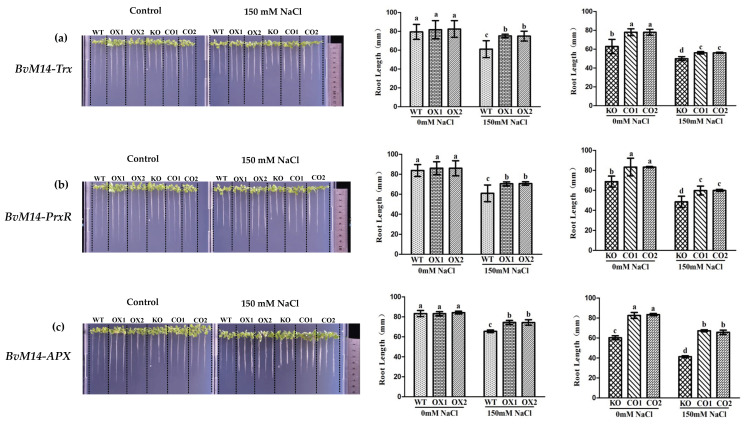
Effects of salt stress on the root length of seedings with different genotypes (WT, OX, KO, and CO): (**a**) Root length of WT and *Trx*-related plants under salt stress; (**b**) root length of of WT and *PrxR-*related plants under salt stress; (**c**) root length of WT and *APX-*related plants under salt stress. Data were analyzed by Duncan’s analysis of variance, and different lowercase letters (a, b, c, d) indicate differences in root length.

Ectopic expression of the six genes encoding the antioxidant enzymes promoted root elongation of transgenic plants under salt stress. With WT plants as control, the root length of plants overexpressing (OX) the *BvM14-Trx*, *BvM14-PrxR*, and *BvM14-APX* increased significantly by 1.3-fold, 1.2-fold, and 1.2-fold after 150 mM NaCl treatment. Compared with knock-out (KO) mutant plants of *Trx*, *PrxR*, and *APX* genes, the root length of complementation transgenic lines (CO) was restored by complementation with the *BvM14-Trx*, *BvM14- PrxR*, and *BvM14-APX* genes to the levels of OX plants (Figure 1). Plants overexpressing other enzyme-coding genes also showed similar phenotypes (Supplementary Figure S1).

**Figure 2:**
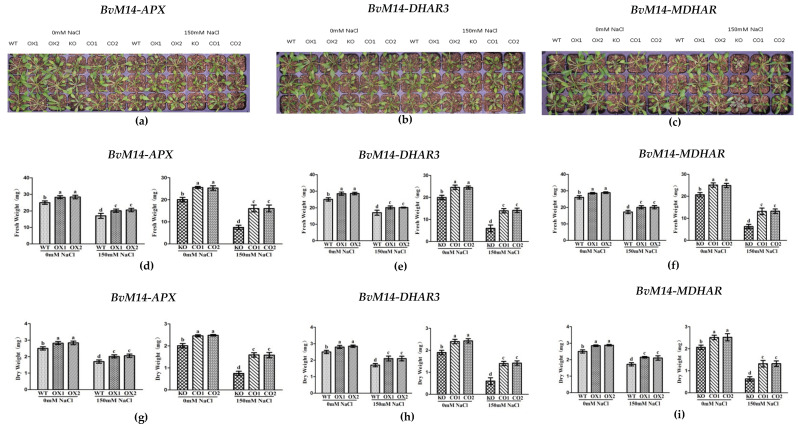
Effects of salt stress on the phenotype and biomass: (**a**) Phenotype of WT and *APX*-related plants under salt stress; (**b**) phenotype of of WT and *DHAR3*-related plants under salt stress; (**c**) phenotype of WT and *MDHAR*-related plants under salt stress; (**d**,**g**) biomass of WT and *APX*-related plants under salt stress; (**e**,**h**) biomass of of WT and *DHAR3*-related plants under salt stress; (**f**,**i**) biomass of WT and *MDHAR*-related plants under salt stress. Data were analyzed by Duncan’s analysis of variance, and different lowercase letters (a, b, c, d) indicate differences in fresh and dry weight.

The overexpression of these six genes increased the fresh and dry weights of the OX plants under salt stress. Compared to the WT plants, the fresh and dry weights of the plants overexpressing *BvM14-APX*, *BvM14-DHAR3*, and *BvM14-MDHAR* genes increased by 1.2-fold, 1.3-fold, and 1.2-fold under 150 mM NaCl stress, respectively, the fresh and dry weights of CO plants were restored, in contrast to the KO plants with mutated *APX*, *DHAR3*, and *MDHAR* genes (Figure 2). Similar phenotypic changes were observed for other OX lines of the antioxidant enzyme-coding genes (Supplementary Figure S2).

### 3.2. Ectopic Expression of Genes Encoding the Antioxidant Enzymes of BvM14 Improved Plant Antioxidant Capacity

**Figure 3:**
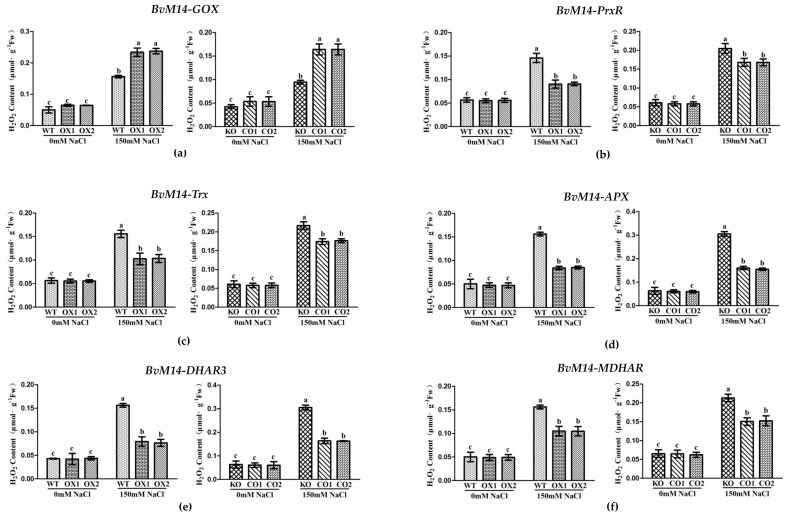
H_2_O_2_ contents of WT and *GOX*, *PrxR*, *Trx*, *APX*, *DHAR3*, and *MDHAR* transgenic plants under salt stress: (**a**) *GOX*-related plants; (**b**) *PrxR*-related plants; (**c**) *Trx*-related plants; (**d**) *APX*-related plants; (**e**) *DHAR3*-related plants; (**f**) *MDHAR*-related plant. Data were analyzed by Duncan’s analysis of variance, and different lowercase letters (a, b, c) indicate differences in H_2_O_2_ contents.

The H_2_O_2_ contents in the WT and OX plants of *BvM14-APX*, *BvM14-DHAR3*, *BvM14-PrxR*, *BvM14-Trx*, and *BvM14-MDHAR* were similar under normal conditions. However, the results became significantly different under salt stress (Figure 3). Interestingly, except for OX plants of *BvM14-GOX*, the rest of the OX plants showed less H_2_O_2_ (Figure 3). Compared to CO lines, the H_2_O_2_ contents in the KO of the genes *APX*, *DHAR3*, *PrxR*, *Trx*, and *MDHAR* showed significantly elevated by1.5-fold, 2.0-fold, 1.2-fold, 1.3-fold, and 1.6-fold under salt stress, respectively.

MDA concentrations were noticeably reduced in *BvM14-DHAR3*, *BvM14-GOX*, and *BvM14-MDHAR* OX compared with WT plants, but there were no significant changes in other OX plants under salt stress conditions, MDA concentrations were significantly elevated in *MDHAR* KO compared with CO plants (Supplementary Figure S3).

**Figure 4:**
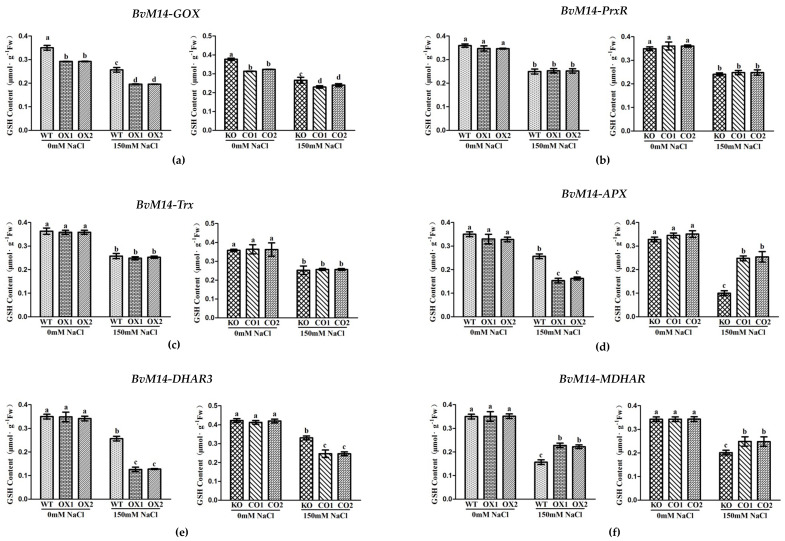
The GSH contents of WT and *GOX*, *PrxR*, *Trx*, *APX*, *DHAR3*, and *MDHAR* transgenic plants under salt stress: (**a**) *GOX-*related; (**b**) *PrxR-*related; (**c**) *Trx-*related; (**d**) *APX-*related; (**e**) *DHAR3-*related; (**f**) *MDHAR-*related plants. Data were analyzed by Duncan’s analysis of variance, and different lowercase letters (a, b, c, d) indicate differences in GSH contents.

The contents of AsA were similar in the *BvM14-Trx*, *BvM14-GOX*, and *BvM14-PrxR* OX plant leaves compared with WT leaves under salt stress. However, the contents of AsA were increased by 1.8-fold and 1.5-fold in the *BvM14-DHAR3* and *BvM14-MDHAR* OX plants, while the content of AsA was decreased by 0.73-fold in the *BvM14-APX* OX plants under salt stress (Supplementary Figure S4). The content of GSH was significantly induced by 1.7-fold in the *BvM14-MDHAR* OX plant leaves compared with WT leaves and reduced by 0.7-fold in the *MDHAR* KO plant leaves compared with CO leaves under salt stress. However, the content of GSH was decreased by 0.6-fold in the *BvM14-DHAR3* OX plants under salt stress. The other transgenic plants did not show significant changes under salt stress (Figure 4).

### 3.3. Antioxidant Enzyme Activities of WT, Different KO, and Transgenic Plants under Salt Stress

**Figure 5:**
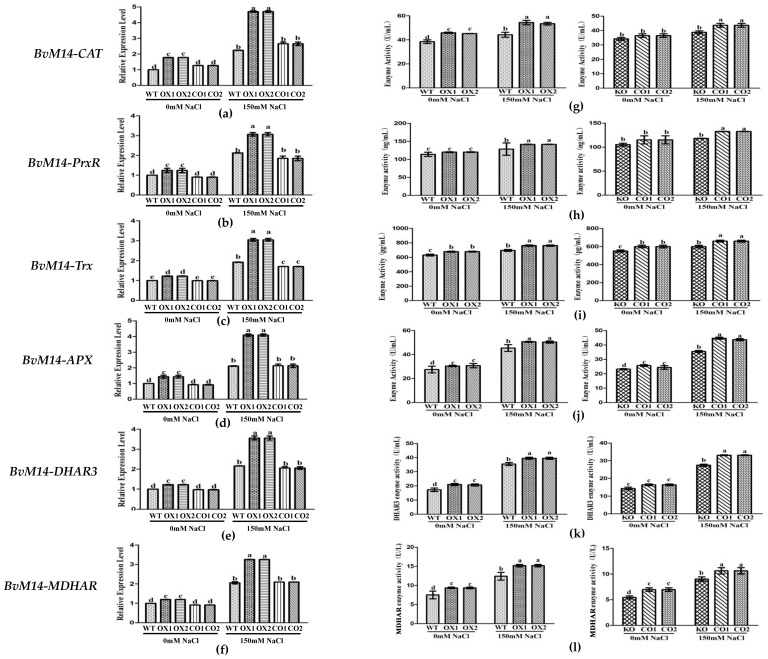
Analyses of relative gene expression and enzyme activities in *GOX*-related plants under salt stress: (**a**–**f**) Expression of antioxidative genes (*BvM14-CAT*, *BvM14-PrxR*, *BvM14-Trx*, *BvM14-APX*, *BvM14-DHAR3*, and *BvM14-MDHAR*) in WT and *GOX*-related plants under salt stress; (**g**–**l**) antioxidant enzyme activities (*Bv*M14-CAT, *Bv*M14-PrxR, *Bv*M14-Trx, *Bv*M14-APX, *Bv*M14-DHAR3, and *Bv*M14-MDHAR) in WT and *GOX*-related plants under salt stress. Data were analyzed by Duncan’s analysis of variance, and different lowercase letters (a, b, c, d, e) indicate differences in gene expression and enzyme activities.

Compared with WT plants, overexpression of *BvM14-GOX* gene increased significantly in gene expression levels and the antioxidant enzyme activities under 150 mM NaCl stress. The Trx and PrxR activities in *BvM14-GOX* gene OX lines were significantly increased by 1.4-fold and 1.2-fold relative to WT under salt stress (Figure 5).

**Figure 6:**
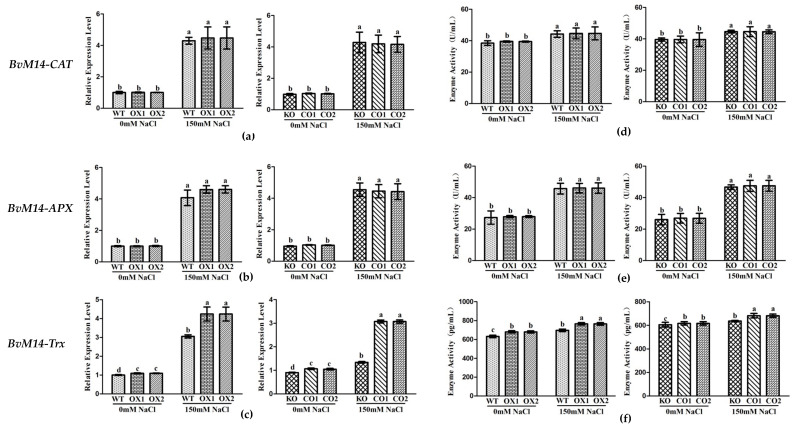
Analyses of relative gene expression and enzyme activities in *PrxR*-related plants under salt stress: (**a**–**c**) Analysis of the expression of antioxidative genes (*BvM14-CAT*, *BvM14-APX*, and *BvM14-Trx*) in WT and *PrxR*-related plants under salt stress; (**d**–**f**) antioxidant enzyme activity (BvM14-CAT, BvM14-APX, and BvM14-Trx) in WT and *PrxR*-related plants under salt stress. Data were analyzed by Duncan’s analysis of variance, and different lowercase letters (a, b, c, d) indicate differences in gene expression and enzyme activities.

The Trx and PrxR enzyme activities in *BvM14-PrxR* gene OX lines and *BvM14-Trx* gene OX lines were significantly higher by 1.5-fold and 1.3-fold than WT under salt stress, respectively (Figure 6 and Supplementary Figure S5). Therefore, it is inferred that the two genes have synergistic effects in plant tolerance to salt stress.

**Figure 7:**
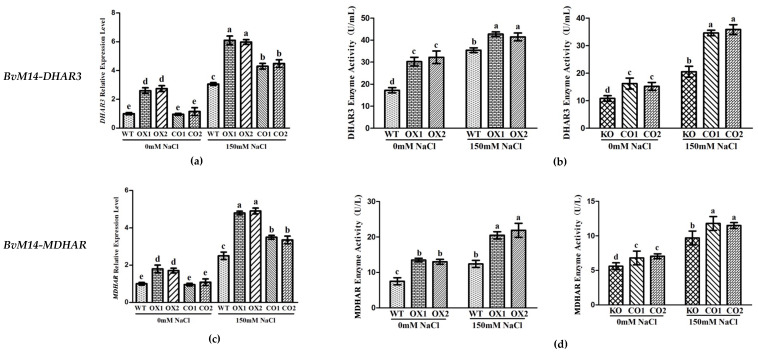
Analyses of relative gene expression and enzyme activities in *APX*-related plants under salt stress: (**a**,**c**) Analysis of the expression of antioxidative genes (*BvM14-DHAR3* and *BvM14-MDHAR*) in WT and *APX-*related plants under salt stress; (**b**,**d**) antioxidant enzyme activity (BvM14-DHAR3 and BvM14-MDHAR) in WT and *APX*-related plants under salt stress. Data were analyzed by Duncan’s analysis of variance, and different lowercase letters (a, b, c, d, e) indicate differences in gene expression and enzyme activities.

In *BvM14-APX* OX lines, the MDHAR and DHAR3 enzyme activity was positively correlated with that of the APX, and both increased by 1.9-fold and 1.3-fold under salt stress. The *MDHAR* and *DHAR3* gene expression levels was increased by 2.3-fold and 2.1-fold in *BvM14-APX* OX lines, and both reduced by 0.6-fold and 0.6-fold in *APX* KO lines under salt stress (Figure 7).

Both in *BvM14-DHAR3* and *BvM14-MDHAR* OX lines, the APX enzyme activity was significantly higher by 1.5-fold and 1.3-fold than WT, in *DHAR3* and *MDHAR* KO lines, the APX enzyme activity was significantly reduced by 0.3-fold and 0.7-fold relative to CO under salt stress (Supplementary Figure S6). Increased DHAR and MDHAR activities were reported in different plants subjected to abiotic stresses [24,25,26].

### 3.4. Different Levels of Regulations of the Six Antioxidant Enzymes of BvM14

**Figure 8:**
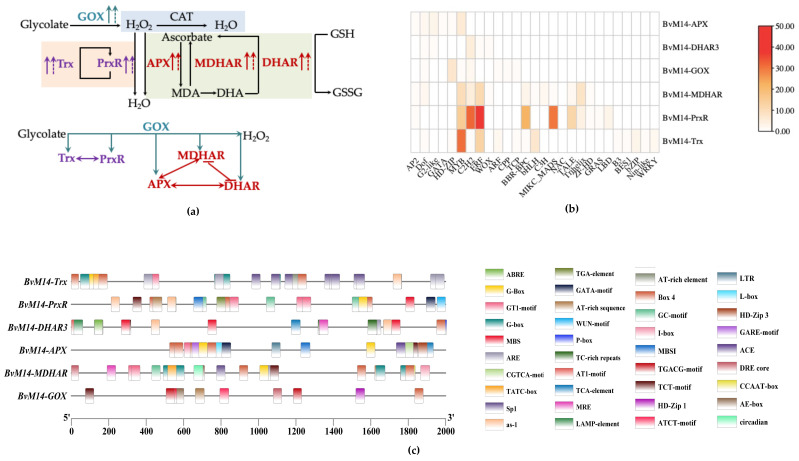
Regulatory networks of the six antioxidant enzymes in the *Bv*M14: (**a**) The role of *BvM14-GOX*, *BvM14-PrxR*, *BvM14-Trx*, *BvM14-APX*, *BvM14-DHAR3*, and *BvM14-MDHAR* gene in antioxidant enzyme system; (**b**) the TF binding motifs in the promoter regions of *BvM14-GOX*, *BvM14-PrxR*, *BvM14-Trx*, *BvM14-APX*, *BvM14-DHAR3*, and *BvM14-MDHAR*; (**c**) localization of the more frequent *cis*-acting elements among the *BvM14-GOX*, *BvM14-PrxR*, *BvM14-Trx*, *BvM14-APX*, *BvM14-DHAR3*, and *BvM14-MDHAR* in the promoter regions. The *cis*-regulatory elements presented are labled with different colors and illustrated on the right side. The TATA-box and CAAT-box are not shown. (The dashed line indicates the transcript level and the solid line indicates the activity level; the arrows indicates positive; the lines indicates negative).

Overexpression of *BvM14-GOX* gene significantly increased the expression and activity of Trx and PrxR enzymes in antioxidant system (Figure 5). The *BvM14-Trx* gene expression and enzyme activity showed the same trend in *BvM14-PrxR* transgenic plants. Compared with *BvM14-APX* KO plants, CO plants with *BvM14-APX* gene restored significantly increased *BvM14-DHAR3* gene and *BvM14-DHAR3* gene expression and enzyme activity after 150 mM NaCl stress. In *BvM14-DHAR3* transgenic plants, *BvM14-APX* gene expression and enzyme activity were both increased, the *BvM14-MDHAR* gene expression and enzyme activity were consistent with *BvM14-APX*. The expression of *BvM14-APX* gene and enzyme activity existed positive correlation to *BvM14-MDHAR*, but the expression of *BvM14-DHAR3* gene and enzyme activity showed the opposite trend (Figure 8a).

To understand the potential transcriptional regulatory mechanisms of *BvM14-GOX*, *BvM14-Trx*, *BvM14-PrxR*, *BvM14-APX*, *BvM14-DHAR3*, and *BvM14-MDHAR* genes, we analyzed the presence of *cis*-regulating elements 2000 bp upstream the TSS. The common *cis*-acting elements (such as enhancer element CAAT-box and core promoter element TATA-box) were not shown in the results, the most abundant elements were salt stress-responsive elements, including ABA-responsive element (ABRE), GT-1 motifs that were present in *BvM14-Trx*, *BvM14-PrxR*, *BvM14-APX*, *BvM14-DHAR3*, and *BvM14-MDHAR*. Moreover, we detected hormone-related *cis*-acting elements, including the jasmonate-responsive element (TGACG motif and CGTCA motif), and the salicylic acid-responsive element (TCA element), as widespread among the *BvM14-Trx*, *BvM14-PrxR*, *BvM14-APX*, *BvM14-DHAR3*, *BvM14-GOX*, *BvM14-GOX*, *BvM14-DHAR3*, and *BvM14-APX*. This suggests that these genes are regulated by abiotic stresses and hormones. The binding sites for the salt stress-responsive TFs MYB and APETALA2/ethylene-responsive element binding factors (AP2/ERF) were found in *BvM14-Trx*, *BvM14-PrxR*, and *BvM14-MDHAR3* (Figure 8b).

## 4. Discussion

Based on the previous transcriptome database of *BvM14* under salt stress, the open-reading frames (ORF) of the coding genes *BvM14-GOX*, *BvM14-PrxR*, *BvM14-Trx*, *BvM14-APX*, *BvM14-MDHAR*, *BvM14-DHAR3* encoding major enzymes in the antioxidant system were cloned and analyzed. *Arabidopsis* KO mutants of the homologous genes, CO lines, OX lines, and WT plants were used to characterize the roles of these enzymes in plant salt stress tolerance. Enzyme activity and transcriptional level of six major enzymes in transgenic lines were detected.

ROS (mainly H_2_O_2_ and O_2_^•−^) accumulation can be used as an important indicator for cellular oxidative stress [27]. MDA content can reflect the degree of membrane lipid peroxidation in plant [28]. The results showed that overexpression of the six genes in *Arabidopsis* increased the tolerance of the OX plants to salt stress, which may be attributed to enhanced antioxidante capacity and reduced oxidative damage by decreasing the contents of H_2_O_2_ and MDA and increasing the AsA and GSH contents. Further analysis showed that overexpression of the *BvM14-GOX* led to significant increases in the expression levels and enzyme activities of other key enzymes in the antioxidant enzyme system. DHAR recycles ascorbic acid (AsA), which is then oxidized to form MDHAR. MDHAR is further converted to dehydroascorbate (DHA). AsA is essential to main the cellular redox state under abiotic stresses. MDHAR accompanies APX and scavenges H_2_O_2_ in the mitochondria and peroxisome [29,30,31,32,33,34]. For example, the BvM14-Trx and the BvM14-PrxR mutually promote each other; the expression level and enzyme activity of BvM14-APX were positively correlated with the expression levels and enzyme activities of BvM14-DHAR3 and BvM14-MDHAR, but the expression level and enzyme activity of BvM14-MDHAR were negatively correlated with the expression level and enzyme activity of BvM14-DHAR3; the PrxR/Trx pathway had no significant interaction with the CAT pathway or the AsA/GSH pathway and participated in the plant antioxidant process independently. Based on these results, a salt stress response regulatory network of *BvM14* antioxidant system was constructed.

GOX plays an important role in the glycolate-glyoxylate conversion during photorespiration, which catalyzes the oxidation of glycolate to generate glyoxylate and H_2_O_2_ [35,36,37,38]. BvM14-GOX regulates the activity of other key enzymes in the antioxidant enzyme system and the expression of corresponding genes by catalyzing the production of H_2_O_2_ from glycolic acid, the regulation of H_2_O_2_ may occur in a fluctuating manner because the association-dissociation of GOX and CAT could take place dynamically and transiently in response to environmental stresses or stimuli. Consistently, related research showed that in spite of the constant and high production of ROS caused by the transgenic *GOX* in rice, but it can assist innate antioxidative systems in modulating ionic and redox homeostasis for salt stress tolerance [39], so as to improve the antioxidant capacity of plants and reduce the inhibition of salt stress on plant growth and development [35].

PrxR/Trx pathway-related coding genes *PrxR* and *Trx* have a vital function in cellular antioxidative defense via eliminating excessive ROS [15,40,41,42]. *BvM14-Trx* gene and *BvM14-PrxR* gene act synergistically to eliminate excess of H_2_O_2_ in plants, but they do not participate in MDA and GSH metabolic pathways since OX plants did not show differences in AsA/GSH pathway and CAT pathway. In *Arabidopsis*, *AtTrxh2* overexpressing transgenic plants exhibited higher activities of antioxidant enzymes including peroxidase (POD), catalase (CAT), and superoxide dismutase (SOD), compared with the plants expressing the empty vector control [43]. In tomato, *SlTrxh* enhanced nitrate stress tolerance with decreased oxidative damage by increased antioxidant enzyme activities and interacted with SlPrx [42]. The AsA-GSH cycle is one of the important antioxidant systems in plants [44,45]. APX is involved in the initial step of the AsA-GSH cycle that scavenges excess ROS and protects the plant from salt stress [46,47]. In AsA/GSH pathway, *BvM14-APX* expression and enzyme activity were positively correlated with *BvM14-DHAR3* and *BvM14-MDHAR* expression and enzyme activity, while *BvM14-MDHAR* expression and enzyme activity were negatively correlated with *BvM14-DHAR3* expression and enzyme activity. In tobacco, overexpression of *MnSOD*, *MDHAR*, *DHAR*, and *CAT* in transgenic plants exhibited the improvement of salt tolerance [29,48]. The improved *MnSOD*, *CAT*, *POD*, *APX*, *DHAR*, *MDHAR*, and *GR* expression was also detected in wheat lines and two *Chrysanthemum* species under cold acclimation [49], which was similar with our results. PrxR/Trx pathway has no obvious interaction with CAT pathway and AsA/GSH pathway in plant antioxidant enzymes system.

The distribution and type of CREs in promoters affect the activities and functions of genes. In this study, through a systematic analysis of CRE in the promoter regions of the six genes, we identified various types of CRE (Figure 8c). Related to salt stress, previous studies have shown that abscisic acid (ABA) responsive element binding protein (AREB)/ABRE binding factors (ABFs) in bZIP transcription factors were involved in salt stress [50,51]. Other research indicated that the GT-1 element directly controls the salt response of *OsRAV2*. The study provided a better understanding of the putative functions of *OsRAVs* and the molecular regulatory mechanisms of plant genes under salt stress [52,53]. In this work, we found the CREs in promoters of the six genes can bind many transcription factors, it contains transcription factors (e.g., MYB, C2H2, ERF) related to salt stress (Figure 8b). Recent studies suggested that the AP2/ERF TF family are involved in abiotic stress adaptation [54,55]. Functional analysis of the *SmAP2-17* gene confirmed its role in plant salt tolerance [56]. It has also been demonstrated that C2H2 zinc finger proteins and MYB transcription factors play vital roles in biotic and abiotic stress tolerance [57].

## 5. Conclusions

In conclusion, we characterized the key genes encoding the major antioxidant enzymes of ROS scavenging system in plant salt stress tolerance. In different pathways, the key enzymes synergistically or antagonistically play important role in plant salt stress tolerance. In the PrxR/Trx pathway, the *BvM14-Trx* and the *BvM14-PrxR* mutually promote each other, but the *BvM14-Trx* gene and *BvM14-PrxR* gene do not affect AsA/GSH pathway and CAT pathway. In the AsA/GSH pathway, *BvM14-APX* expression and enzyme activity were positively correlated with *BvM14-DHAR3* and *BvM14-MDHAR* expression and enzyme activity. Meanwhile, the CREs in promoters contain salt stress-responsive elements ABRE, GT-1 motifs and the CREs in promoters of the six genes can bind transcription factor MYB, C2H2, and ERF related to salt stress. Based on the working model, it became clear that multiple levels of regulations, including transcription and translation, are important in controlling plant salt stress tolerance. Future efforts in improving crop salt stress tolerance can benefit from the results from this study and need to consider multiple genes and markers to achieve optimal outcomes.

## Data Availability

The data are contained within the article and supplementary materials.

## Supplementary Materials

The following supporting information can be downloaded at: https://www.mdpi.com/article/10.3390/antiox12010057/s1, Table S1: List of the primer sequences for the six genes tested by RT-PCR; Figure S1: Effects of salt stress on the root length of seedlings with different genotypes (WT, OX, KO, and CO). (a) Root length of WT and *GOX*-related plants under salt stress; (b) Root length of of WT and *DHAR3*-related plants under salt stress; (c) Root length of WT and *MDHAR*-related plants under salt stress; Figure S2: Effects of salt stress on the phenotype and biomass. (a) Phenotype of WT and *Trx*-related plants under salt stress; (b) Phenotype of of WT and *PrxR*-related plants under salt stress; (c) Phenotype of WT and *GOX*-related plants under salt stress; (d,g) Biomass of WT and *Trx*-related plants under salt stress; (e,h) Biomass of of WT and *PrxR*-related plants under salt stress; (f,i) Biomass of WT and *GOX*-related plants under salt stress; Figure S3: MDA content of WT and *GOX*, *PrxR*, *Trx*, *APX*, *DHAR3*, and *MDHAR-*related plants under salt stress; Figure S4: AsA contents of WT and *GOX*, *APX*, *DHAR3*, and *MDHAR*-related plants under salt stress; Figure S5: (a-c) Analysis of the expression of antioxidative genes (*BvM14-CAT*, *BvM14-APX* and *BvM14-PrxR*) in WT and *Trx*-related plants under salt stress; (d-f) Antioxidant enzyme activity (BvM14-CAT, BvM14-APX, and BvM14-PrxR) in WT and *Trx*-related plants under salt stress; Figure S6: Analyses of relative gene expression and enzyme activities in *DHAR3*-related and M*DHAR*-related plants under salt stress. (a,c) Analysis of the expression of antioxidative genes (*BvM14-APX* and *BvM14-MDHAR*) in WT and *DHAR3*-related plants under salt stress; (b,d) Antioxidant enzyme activity (BvM14-APX and BvM14-MDHAR) in WT and *DHAR3*-related plants under salt stress; (e,f) Analysis of the expression of antioxidative genes (*BvM14-MDHAR* and *BvM14-APX*) in WT and M*DHAR*-related plants under salt stress; (g,h) Antioxidant enzyme activity (*Bv*M14-MDHAR and *Bv*M14-APX) in WT and M*DHAR*-related plants under salt stress; Figure S7: Identification of overexpression of *BvM14-GOX*, *BvM14-PrxR*, *BvM14-Trx*, *BvM14-APX*, *BvM14-DHAR3*, *BvM14-MDHAR* and *atpox*, *atprxr*, *attrx*, *atapx*, *atdhar3*, *atmdhar* mutant in *Arabidopsis*. (a) qRT-PCR analysis of the expression levels of the overexpressed *BvM14-GOX* (OX1 and OX2) in *Arabidopsis*; (b) qRT-PCR analysis of the expression levels of *AtPOX* in the *atpox* mutant; (c) qRT-PCR analysis of the expression levels of the overexpressed *BvM14-PrxR* (OX1 and OX2) in *Arabidopsis*; (d) qRT-PCR analysis of the expression levels of *AtPrxR* in the *atprxr* mutant; I qRT-PCR analysis of the expression levels of the overexpressed *BvM14-Trx* (OX1 and OX2) in *Arabidopsis*; (f) qRT-PCR analysis of the expression levels of *AtTrx* in the *attrx* mutant; (g) qRT-PCR analysis of the expression levels of the overexpressed *BvM14-APX* (OX1 and OX2) in *Arabidopsis*; (h) qRT-PCR analysis of the expression levels of *AtAPX* in the *atapx* mutant; (i) qRT-PCR analysis of the expression levels of the overexpressed *BvM14- DHAR3* (OX1 and OX2) in *Arabidopsis*; (j) qRT-PCR analysis of the expression levels of *AtDHAR3* in the *atdhar3* mutant; (k) qRT-PCR analysis of the expression levels of the overexpressed *BvM14-MDHAR* (OX1 and OX2) in *Arabidopsis*; (l) qRT-PCR analysis of the expression levels of *AtMDHAR* in the *atmdhar* mutant.
