# Supplementary material for: Functional Characterization of Sugar Beet M14 Antioxidant Enzymes in Plant Salt Stress Tolerance
Source: Antioxidants (Basel). 2022 Dec 27;12(1):57. doi: 10.3390/antiox12010057 (PMC9854869; doi:10.3390/antiox12010057)

BvM14-DHAR3 transgenic lines

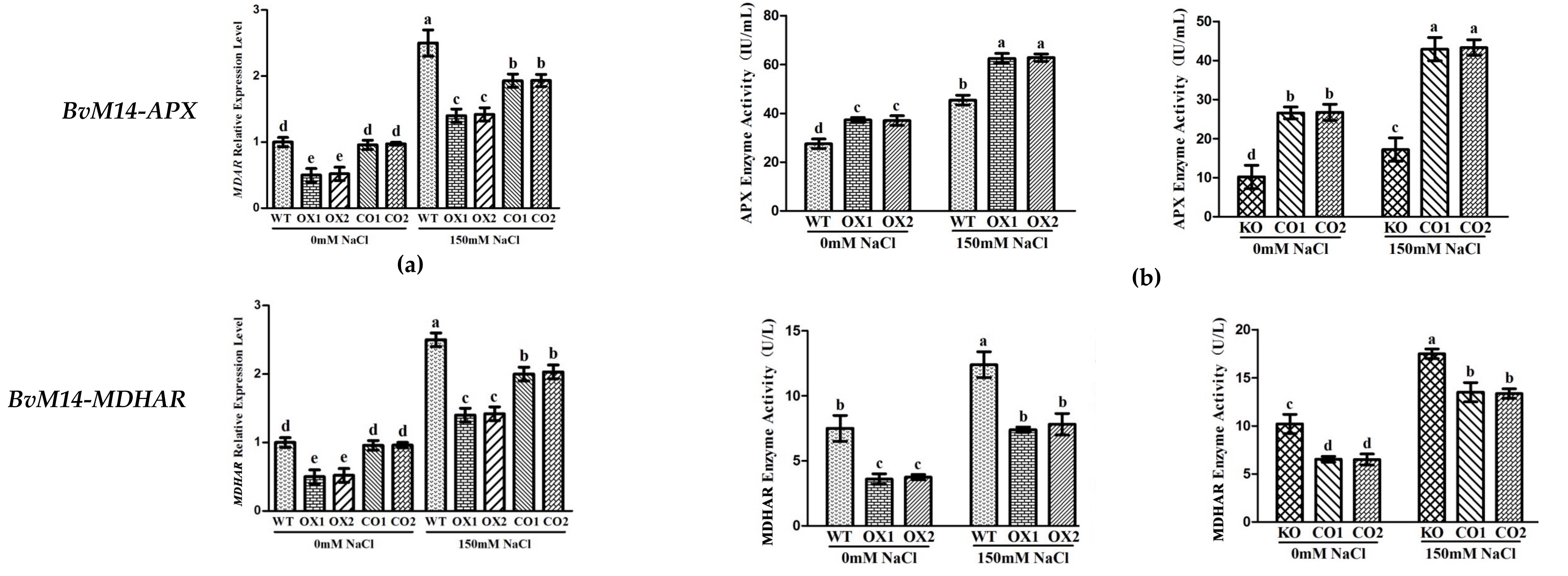

BvM14-MDHAR transgenic lines

(c) (d)

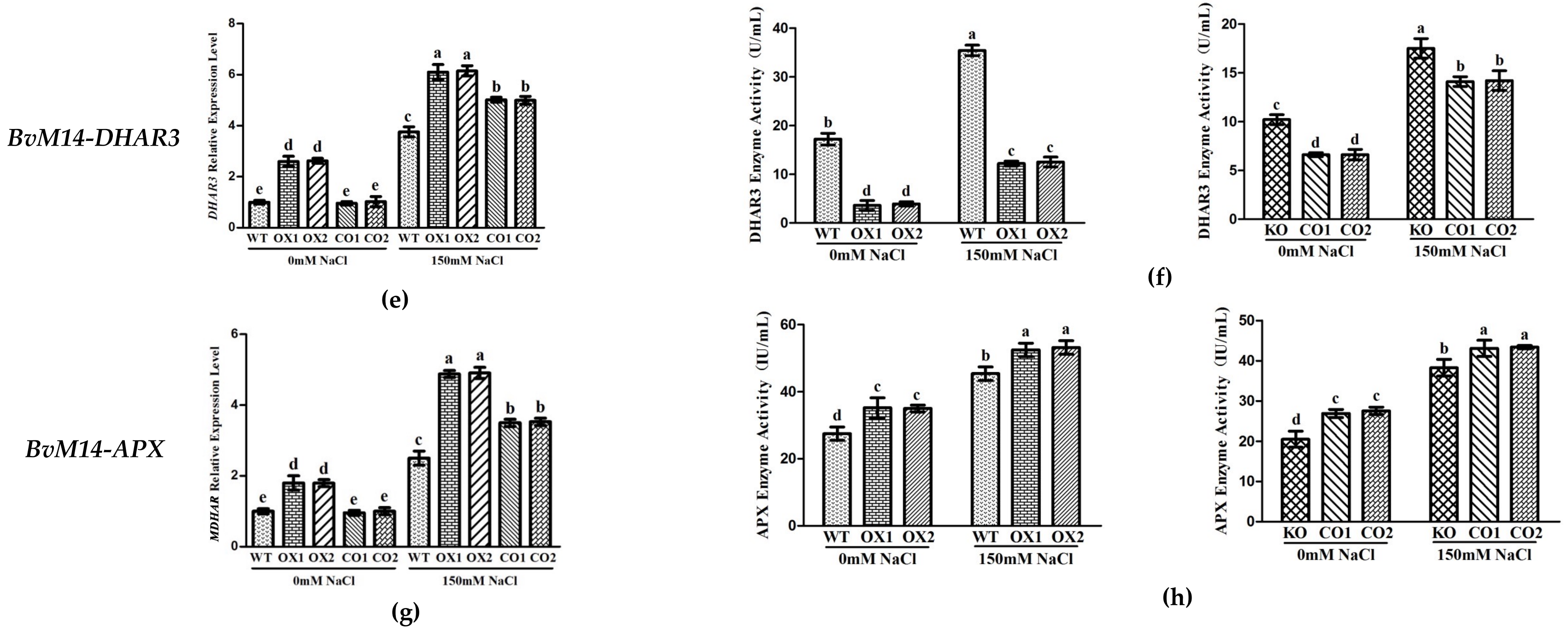

Supplement: Supplementary file 1 [file antioxidants-12-00057-s001.zip › Figure S6.pdf]
