# Supplementary material for: Functional Characterization of Sugar Beet M14 Antioxidant Enzymes in Plant Salt Stress Tolerance
Source: Antioxidants (Basel). 2022 Dec 27;12(1):57. doi: 10.3390/antiox12010057 (PMC9854869; doi:10.3390/antiox12010057)
Supplement: Supplementary file 1 [file antioxidants-12-00057-s001.zip › Table S1.pdf]

Gene name	Primer sequence
BvM14-DHAR3	*BvM14-DHAR3*s：*5′-ATA GGC AAA GGC ACA TCA AAA CG-3′*<br>*BvM14-DHAR3as*：*5′-CCA CTA CAT GAC TAC CTG CAA ATC C-3′*<br>*BvM14-DHAR3s*：*5′-CGGTTCCTGACTCACTTCC-3′*<br>*BvM14-DHAR3as*：*5′-CAGCCAGCAACGACATCC-3′*
BvM14-MDHAR	*BvM14-MDHARs*：*5′-TTT CTC TCT CAT CCT TCT TTC TCT G-3′*<br>*BvM14-MDHARas*：*5′-ATT CTC AAT GTC ATC CAC ACA CTA C-3′*<br>*BvM14-MDHARs*：*5′-CTGATATTGTGATTGTTGGTGTTG-3′*<br>*BvM14-MDHARas: 5′-AAGGCATCGGTCTTGATTCC-3′*
BvM14-APX	*BvM14-APXs*：*5′-CGC GGA TCC TCA CTC TCC AAT TTC TAG GGT TC-3′*<br>*BvM14-APXas*：*5′-CCG CTC GAG CCT ACT ACA CAT TCC AAT CCA TCA C-3′*<br>*BvM14-APXs*：*5′-GTATGGAAGAGTGGAGGTGAC-3′*<br>*BvM14-APXas*：*5′-TAGATGTTGAGCAGGTGAAGG-3′*
BvM14-Trx	*BvM14-Trxs*：*5′-TTTCCTTGCTCAACACTAC-3′*<br>*BvM14-Trxas*：*5′-CAGCAACTTACTTAAGAGCCAG-3′*<br>*BvM14-Trxs*：*5′-ACAAGTTGAACACAGATGAATCC-3′*<br>*BvM14-Trxas*：*5′-GCACAGCACCAATAATACTCTC-3′*
BvM14-PrxR	*BvM14-PrxRs*：*5′-ATTCTTCAATGGCCCTCT-3′*<br>*BvM14-PrxRas*：*5′-AGAAGGGATAAAGGATTCACAG-3′*<br>*BvM14-PrxRs*：*5′-GGGAAGCCTGTTGTTGTTTAC-3′*<br>*BvM14-PrxRas*：*5′-TCATCACCACTAATACCAATAACC-3′*
BvM14-GOX	*BvM14-GOXs*：*5′- GAGATGGAGGTCACCAATGTCAACG-3′*<br>*BvM14-GOXas*：*5′- TTATAGCTTCCATTATTTGTGCACAG-3′*<br>*BvM14-GOX-RTs*：*5′-TGGCACTGAGCGGTTGTC-3′*<br>*BvM14-GOX-RTas*：*5′-CGAGAAGAGCGAGGAGCATC-3′*
