# Supplementary figures and images for: Functional Characterization of Sugar Beet M14 Antioxidant Enzymes in Plant Salt Stress Tolerance
Source: Antioxidants (Basel). 2022 Dec 27;12(1):57. doi: 10.3390/antiox12010057 (PMC9854869; doi:10.3390/antiox12010057)

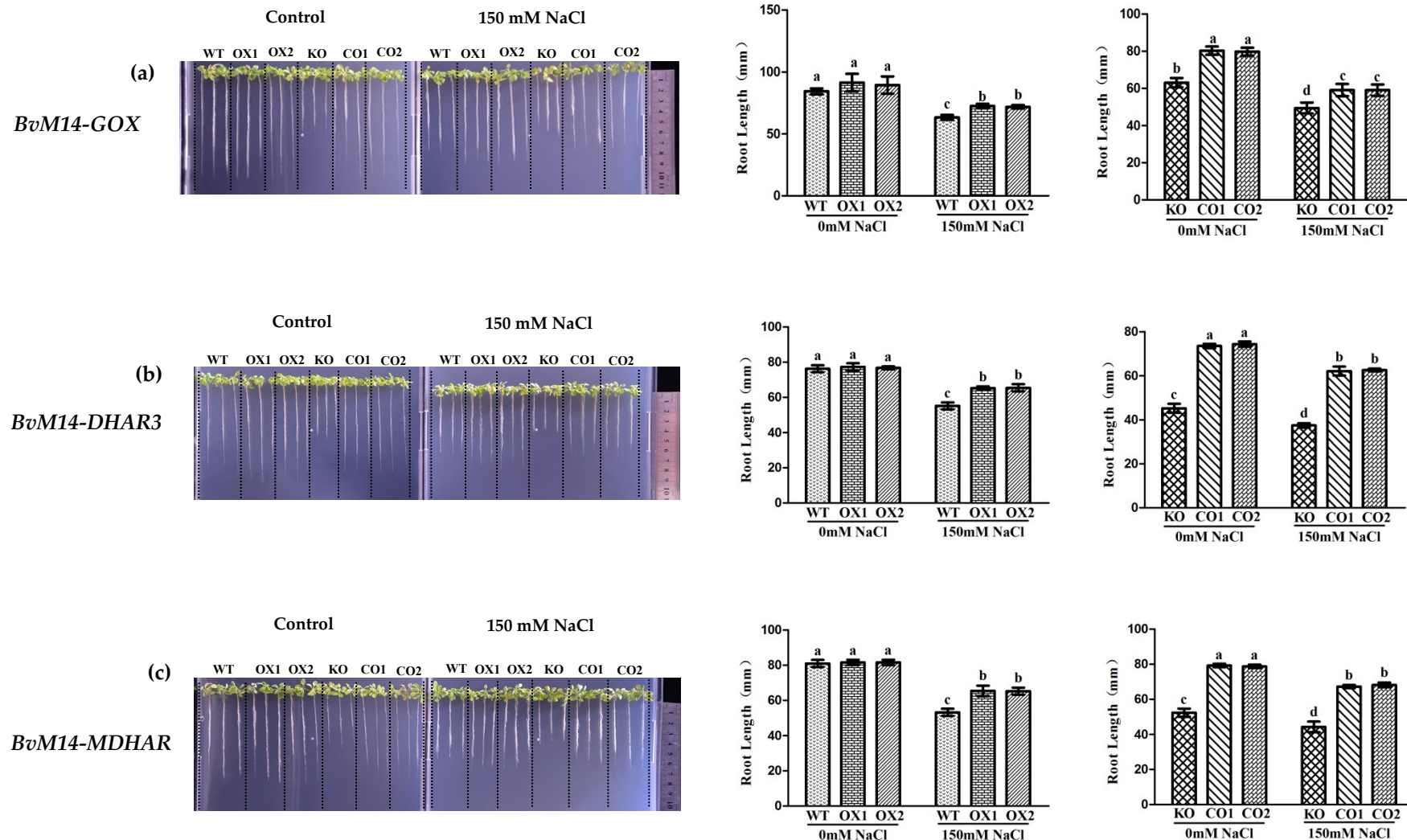

Figure S1

Supplement: Supplementary file 1 [file antioxidants-12-00057-s001.zip › Figure S1.pdf]

*BvM14-Trx*

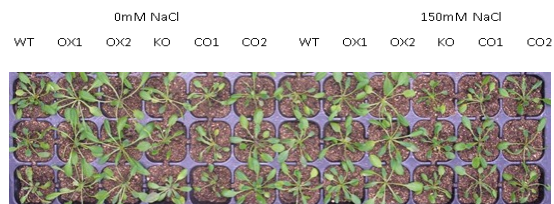

(a)

*BvM14-PrxR*

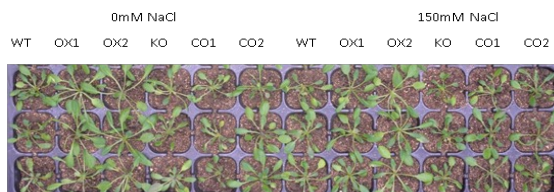

(b)

*BvM14-GOX*

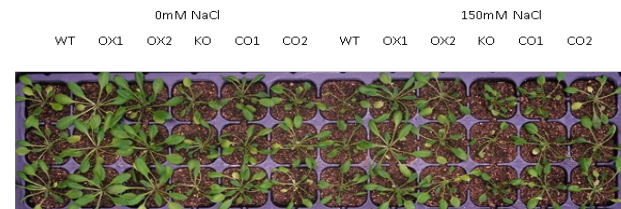

(c)

*BvM14-Trx*

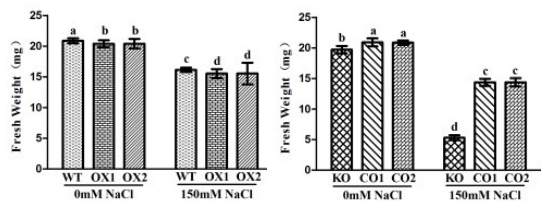

(d)

*BvM14-PrxR*

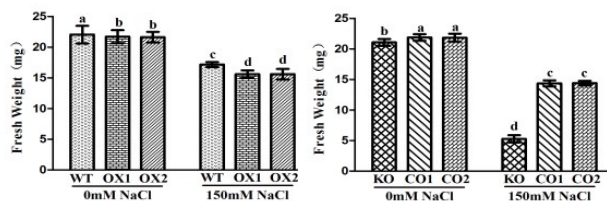

(e)

*BvM14-GOX*

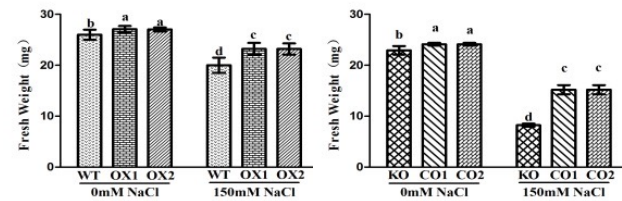

(f)

*BvM14-Trx*

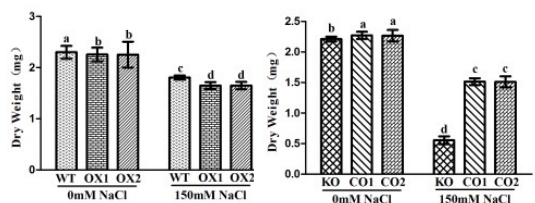

(g)

*BvM14-PrxR*

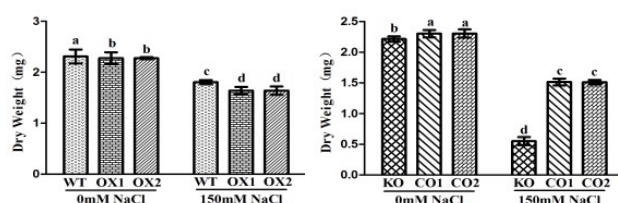

(h)

*BvM14-GOX*

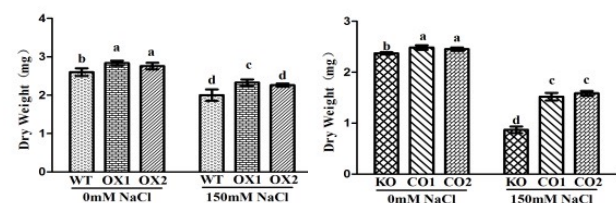

(i)

Figure S2

Supplement: Supplementary file 1 [file antioxidants-12-00057-s001.zip › Figure S2.pdf]

*BvM14-GOX*

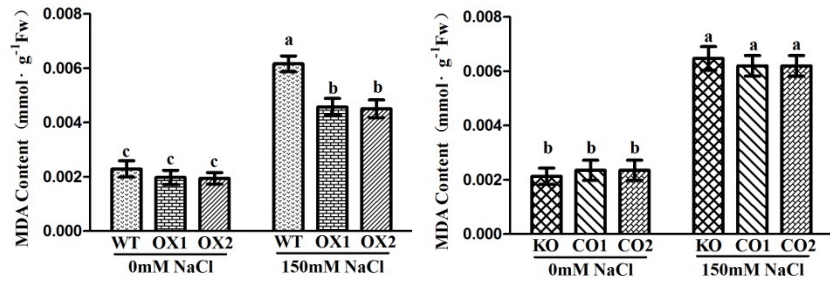

(a)

*BvM14-PrxR*

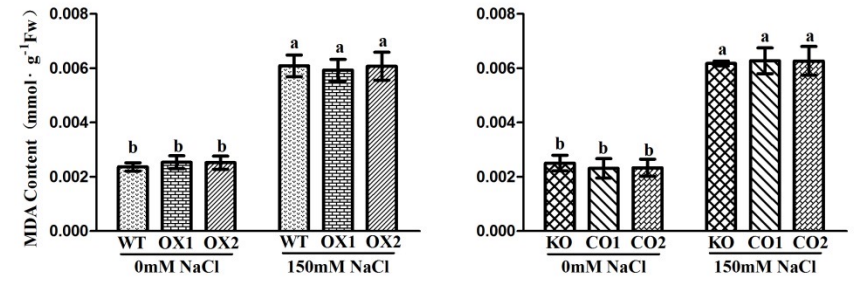

(b)

*BvM14-Trx*

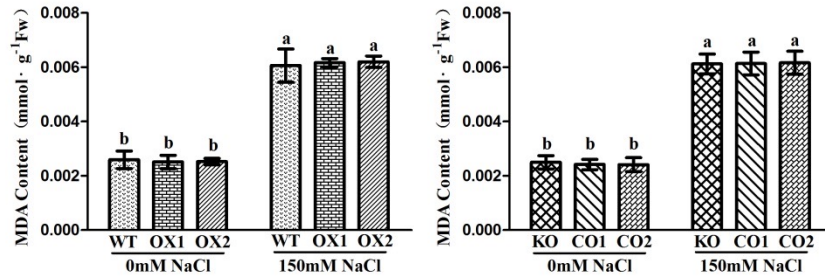

(c)

*BvM14-APX*

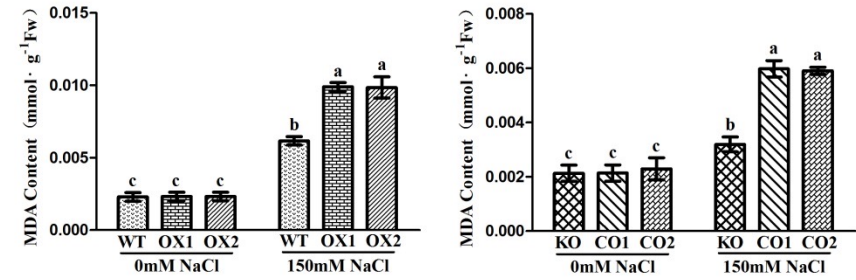

(d)

*BvM14-DHAR3*

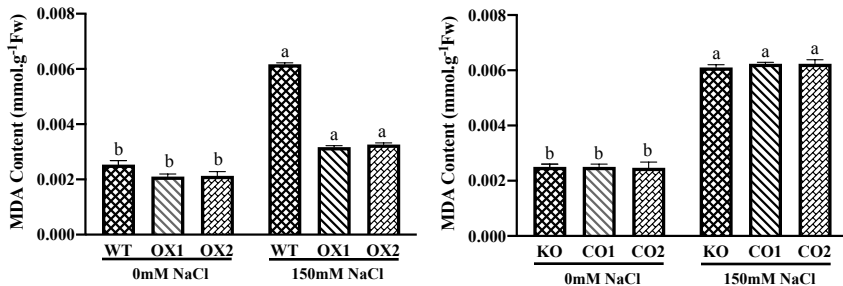

(e)

*BvM14-MDHAR*

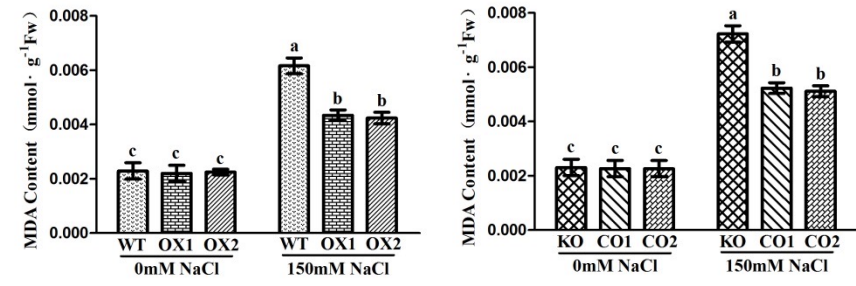

(f)

Figure S3

Supplement: Supplementary file 1 [file antioxidants-12-00057-s001.zip › Figure S3.pdf]

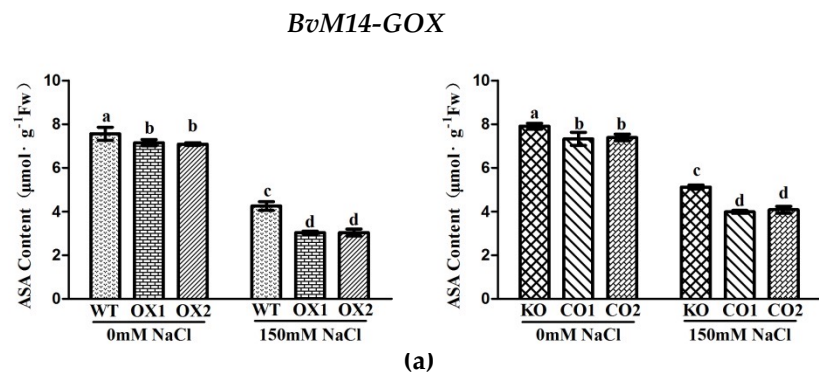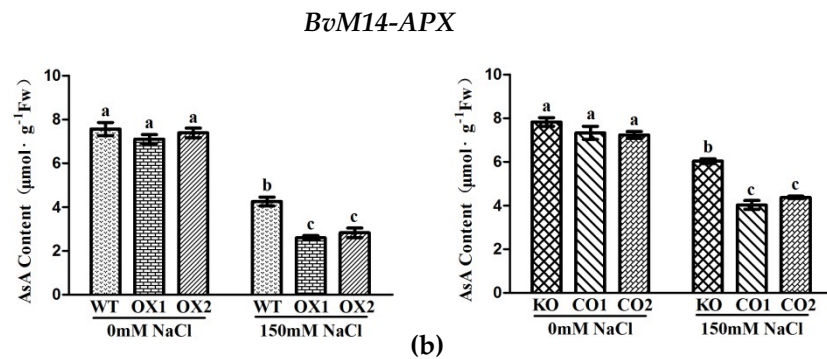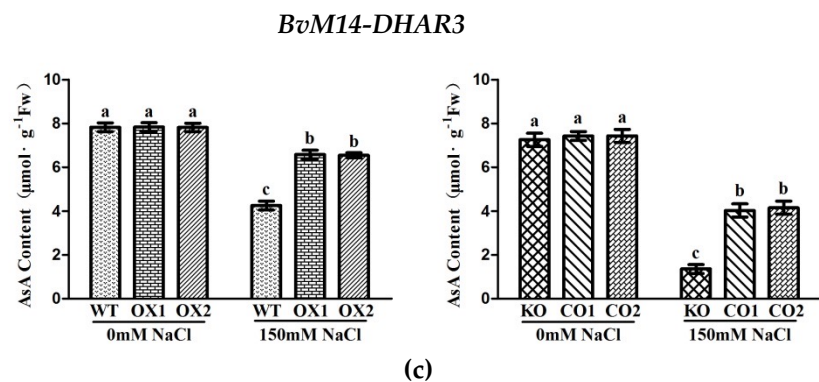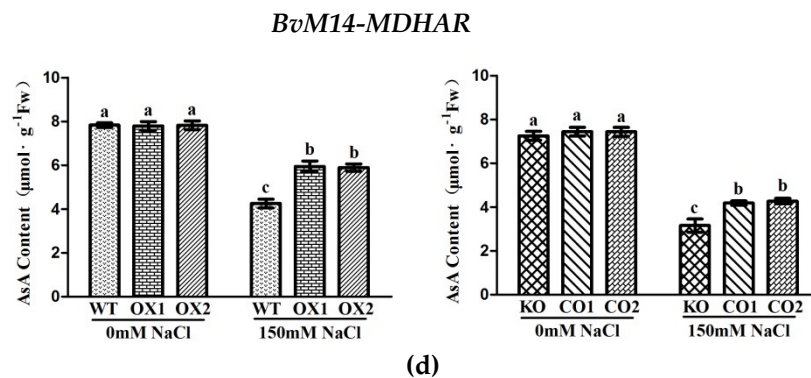

Figure S4

Supplement: Supplementary file 1 [file antioxidants-12-00057-s001.zip › Figure S4.pdf]

### *BvM14-Trx* transgenic lines

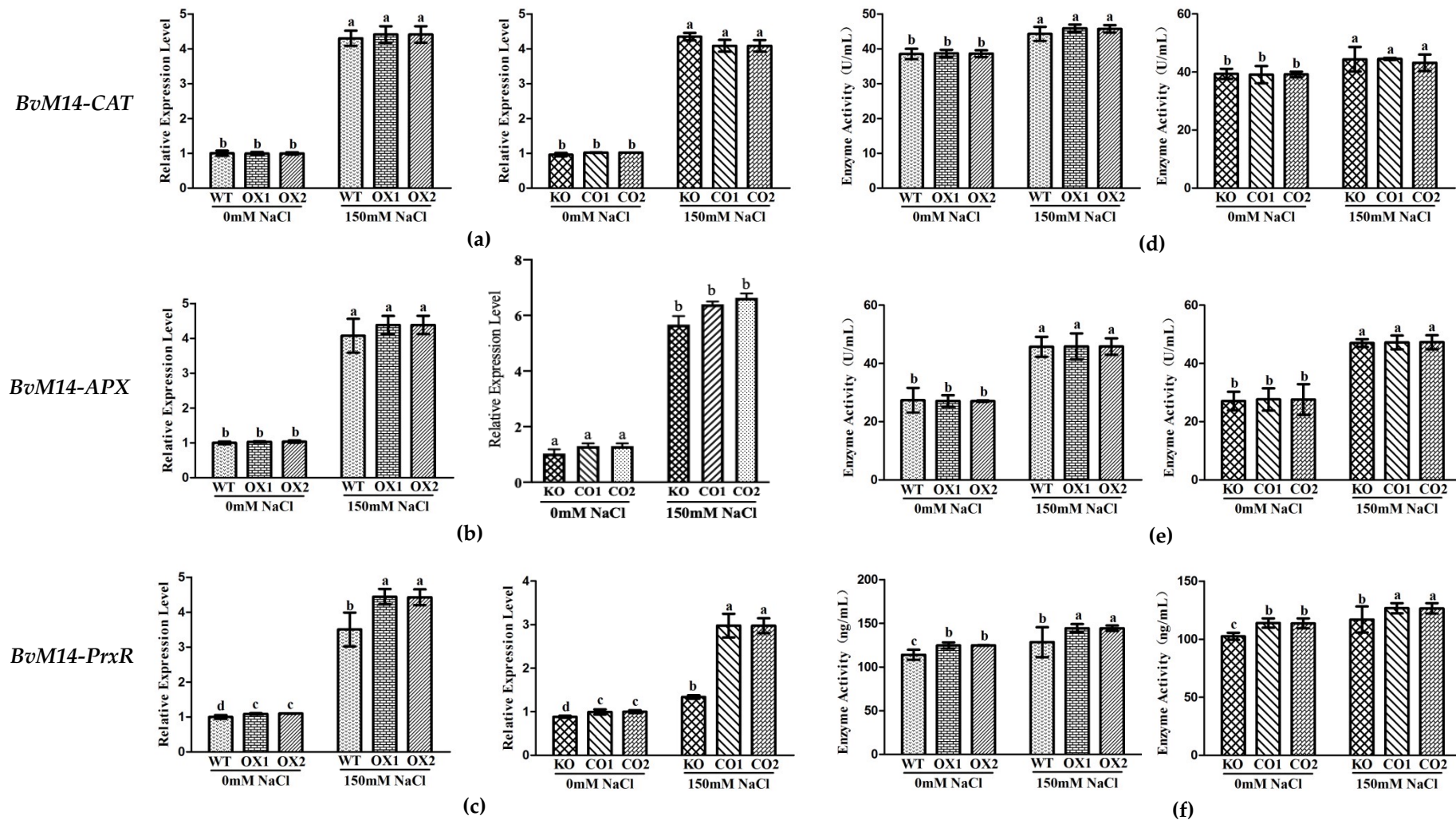

Figure S5

Supplement: Supplementary file 1 [file antioxidants-12-00057-s001.zip › Figure S5.pdf]

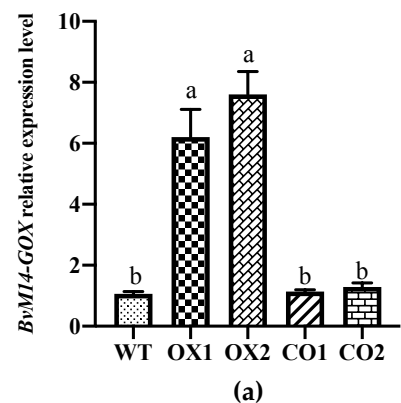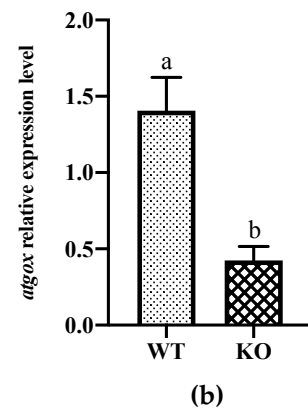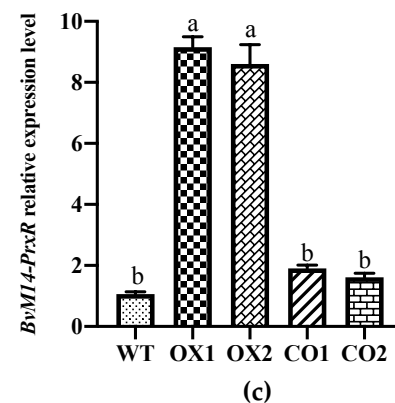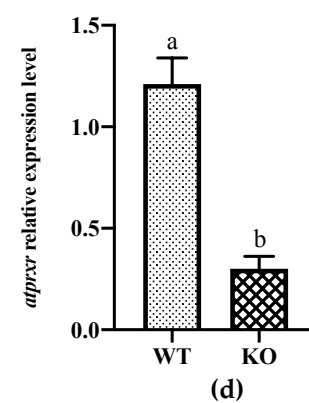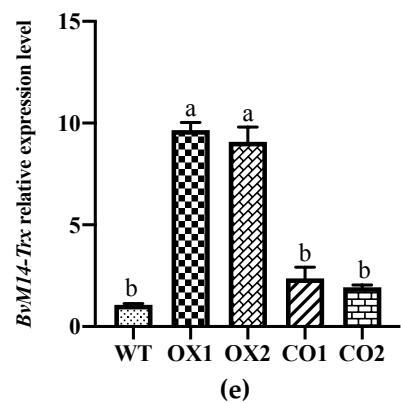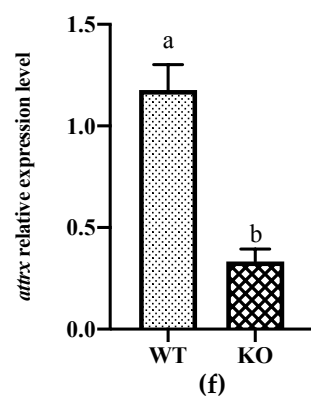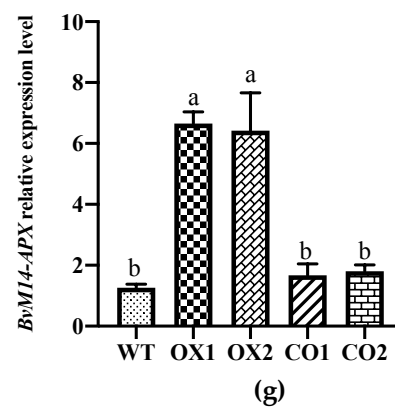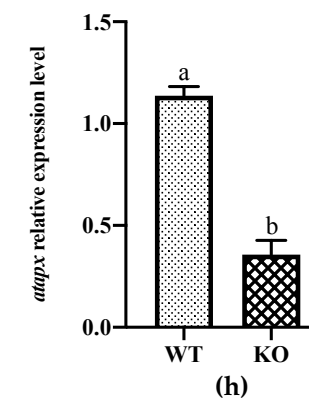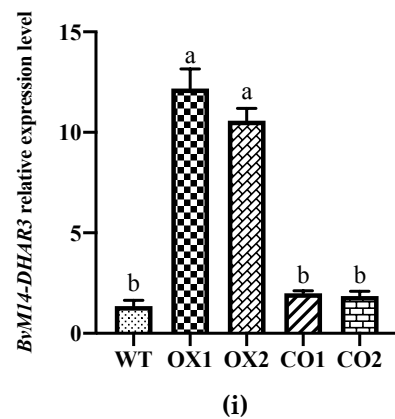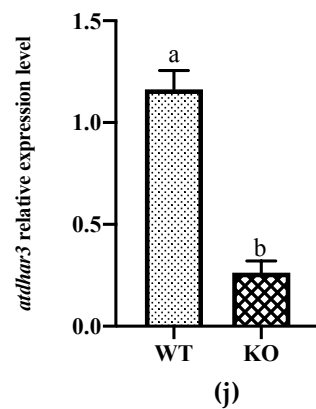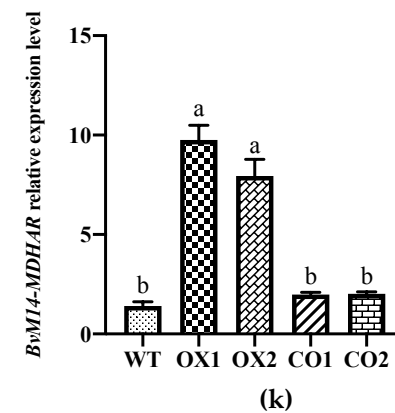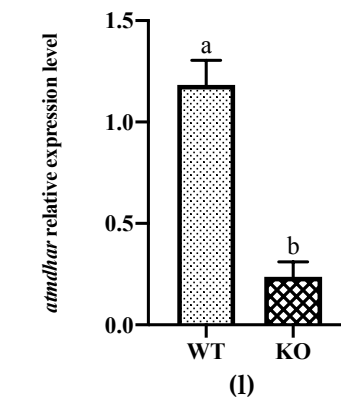

Figure S7

Supplement: Supplementary file 1 [file antioxidants-12-00057-s001.zip › Figure S7.pdf]
